# Diphtheria

**Published:** 1897-11

**Authors:** Gardner T. Swarts


### International Dental Journal.

Vol. XVIII. November, 1897. No. 11.

### Original Communications.[1]

### DIPHTHERIA.[2]

BY GARDNER T. SWARTS, M.D.[3]

I feel, of course, highly honored to speak to you on any subject whatever, but feel especially pleased to think that gentlemen in your line of work should devote some time to learning something about a disease in which I have been especially interested. Of course, men in the medical profession should be specially interested in all diseases, but the question of typhoid fever and this subject of diphtheria are topics that are of especial importance to us, because of the great number of human lives involved.

I am sorry on account of the regularity in the publication of your transactions that I have no paper. My apology for not presenting one is that the subject, being rather dry, the reading of a prepared paper might be recommended as a cure for insomnia, and that is not the effect which I wish to obtain this evening.

The word "diphtheria" comes from the Greek, and means "a tanned hide." The reason for that, I presume, is the resemblance of the characteristic form and texture of the membrane which is

[1] The editor and publishers are not responsible for the views of authors o papers published in this department, nor for any claim to novelty, or otherwise, that may be made by them. No papers will be received for this department that have appeared in any other journal published in the country.

[2] Read before the American Academy of Dental Science, March 3, 1897.

[3] Secretary of the Rhode Island State Board of Health.

found in this disease, which we have called diphtheria. As we come later to the clinical symptoms, we will find that this analogy is very close.

Diphtheria, as the disease diphtheria, may have been with us for centuries, but that claim is disputed by many able men, especially the older practitioners, who practised back in the fifties, who give us the historical information that this disease, with this peculiar formation of membrane, did not make its appearance in the United States until about the year 1857 ; that previous to that time we had sore throats, tonsillitis, pharyngitis, laryngitis, quinsy,—we had various formations in the throat and inflammatory conditions there, but nothing like that which we now characterize as diphtheria and with which peculiar constitutional symptoms occur. This disease in the production of its symptoms is very characteristic, and when a typical case occurs is not mistaken for any of the other conditions ; but, unfortunately for the patient as well as for the physician, those constitutional symptoms, by which we can determine whether the disease is or is not diphtheria, very often do not present themselves until possibly we are about ready to sign the death certificate. In fact, it has, up to within two or three years, been a common saying among physicians that if a person presented himself to a physician with a tonsillitis or an ordinary sore throat and he wanted to make sure of his diagnosis, the better way would be to have his treatment follow pretty closely the treatment he would prescribe for diphtheria, so you can understand how he has been at sea with regard to this disease. When he thought he was treating possibly a case of tonsillitis, the patient would die, and that would be the first intimation that the physician received that the patient was suffering from diphtheria. Of course, that was very unfortunate for the case and for the physician as well.

When a case of diphtheria appears it commonly presents itself as a simple sore throat. Usually for true diphtheria, uncomplicated with any local inflammatory condition, the symptoms are not very well marked. Even children, who are apt to have it pretty severely, will make little or no complaint in its earlier stages, and adults will treat the case as just a simple sore throat, whereas in many of the less dangerous diseases of the throat, especially follicular tonsillitis, the pain and distress occasioned by swallowing are so great that the patient's alarm is proportionately great, and this peculiar condition has to be borne in mind in looking for the symptoms. Of course, the temperature and pulse are also to be considered, but here again we find another difficulty. In true diphtheria we find a rise of temperature of not over 101° or 102° F., whereas in a simple follicular tonsillitis, occurring in some people every fall, the temperature will run up to 103° or 104°, or even 105°, giving alarm to the patient and the physician. This is one of the points of distinguishing between the malignant disease and the diseases which are not dangerous. If, in looking in upon the throat, the fauces are found to be more or less reddened, the tonsils possibly bulging out and excreting a certain amount of fungus, and there is generally a great deal of inflammation and corresponding distress to the patient, we may feel, from the clinical examination, that we are dealing with a case of simple pharyngitis; but, however trifling the inflammation may appear, if we discover in connection with it a slimy white patch, with a firm consistency, whether it be the size of a pinhead or whether it cover the whole of the pharynx, and whether the patient is much or little distressed, clinically, we are justified in the belief that it is diphtheria. We find a white patch in other oral and throat-diseases, but in those cases the membrane is soft and easily pushed aside. If it is true diphtheria, this false membrane, when removed from the throat forcibly, is found to be a tough, a tenacious, thick, clinging membrane, which has been described as a "leathery patch," which is removed with some difficulty, and leaves a denuded, bleeding membrane behind. This is not the case with the soft membranes which are found in the other diseases which are allied to this.

The symptoms which we look for, then, are more or less inflammation in the throat, more or less rise of temperature, more or less depression of the heart, and the presence of the tough, white membrane. There we have, first and last, the clinical symptoms that are present in cases of diphtheria; but, as I have stated, many of these symptoms being present in other diseases, we have chiefly depended in our diagnosis upon finding the actual typical membrane of diphtheria. We have learned a great deal in the last two or three years, and this knowledge has been brought about by the study of bacteriology, and particularly since Klebs and Löffler, a Frenchman and a German, in their combined investigations, discovered a microorganism, a bacterium, which was always found when this tough, thick membrane was present, and even when it was not present, when the symptoms were as severe as I have described with regard to the extreme depression of the system. This organism was called the Klebs-Löffler bacillus, and at the present time it is the seeking out and finding of that organism on which we depend for our diagnosis instead of depending upon finding the typical membrane. We have found that it makes up the greater amount of this white membrane, and these bacilli, finding suitable food in the mucous membrane of the throat, become firmly lodged there, thus causing the bleeding which happens when the membrane has been removed. In studying out the manner of growth of these organisms, it is found that oftentimes the patient will exhibit all the constitutional symptoms of the disease and yet the membrane may not form, so we are unable at the present time by examining the throat to determine if the bacillus is present or is absent. If the white membrane is present, we look out for mischief; if it is absent, it is not positive proof that the organism is not there. Neither does the amount of inflammation present assist us, except that we may conclude that those cases which seem to be most alarming clinically are probably not diphtheria. It is not enough that the organism finds its way into the throat to produce diphtheria, it must also find in the throat an inflamed condition which will cause the exudation of serum and be a suitable nutrient media, an agreeable soil on which it can grow. If it falls upon a healthy throat, it is like grains—oats, wheat, or other grains—which fall upon rocks, they will not grow, even under the most favorable climatic conditions, whereas if they fell upon suitable soil each grain would duplicate itself many hundred times. So it is with these organisms. While they may be present in the mouth, they will not multiply unless the conditions are favorable, and it is because of this that many persons who are not suffering from diphtheria themselves go about disseminating these organisms in the various methods, of which I will speak later, causing the mischief which we call epidemic.

Now, the ways in which this disease can be transmitted are very numerous. Probably the most common way is by means of a common drinking-cup, or using cups and glasses that others have used. In talking to you, I am proceeding on the assumption that you all believe that the organism may produce the disease, and therefore shall not go into the details of the bacteriological investigations any further than to say that the fact that some diseases may be transmitted from one person to another is proved by showing that the bacillus can be taken from the body, can be grown in a suitable media, and can be reproduced in animals or other persons, with all the constitutional symptoms of the original disease. This, of course, it is possible to do with diphtheria. As I say, this organism in a healthy throat does not produce the disease always, but is very liable to in a throat where there is any inflammation, and it can easily be seen that the drinking-cup is one of the most direct means of transmission from one individual to another; and this has led, as you have probably learned, in several churches to the adoption in the communion service of individual drinking-cups instead of the common drinking-cup. There is a great deal of opposition to this, many claiming that it is not a communion unless from the same cup; but the claim goes back that the pitcher from which it is poured is the common cup, consequently the distinction cannot hold. The method, which was first adopted in Brooklyn, I believe, and is now used in some of the New York churches, as well as other churches through the country, is to place these individual cups on trays holding say twenty or thirty, and these are passed about among the congregation with great celerity, and the whole practice is carried on more quickly than by using the common cup. As an illustration of the effect of the use of the common cup method, a case is cited of a lady in Providence who was prevented from participation in the communion service each Sunday, to her very great regret, by the fact that there sat in a pew just in front of her an old gentleman who had a carcinoma of the lip, and she could not bring herself to drink from the cup which was offered to her directly after he had used it; so really the use of the individual cup would bring her perhaps in closer communion with the congregation than the other method. Other means of communication are familiar to the health departments of the various cities, especially among children. One reason, perhaps, why the disease is more common among children is that their playthings are often used in common. You can readily conceive how the use of a fish-horn passed from mouth to mouth may be a means of transmission of various diseases. And then in the public schools, the books and slates and pencils are used in common, and mouthing the pencils is a direct means of transmission. I can give you a good illustration of one of the simple ways by which the disease may be effectually transmitted. I was called to a case of diphtheria, and found a child moribund, or who would be within twelve hours, and the only case of diphtheria in the neighborhood was a child in the same house, who had had proper treatment and had recovered from the disease, so that the case was one which, before the advent of bacteriology, would have been very difficult to explain. The dying one had been away from the house until the child who had been sick was pronounced entirely free from diphtheria and the house had been fumigated, and it was thought there could be no more danger from the disease. Perhaps, if circumstances had not come about as they did, there would have been no danger; but it so happened that the child who had been sick had a ring on its finger which it could not get off. Now, you know, the way to remove a ring that fits tightly on the finger is to suck the finger and moisten it, so in this case number one sucked the finger and did not succeed in getting it off, and then number two tried it, and you can see that the child who was apparently fully recovered from diphtheria was making a direct inoculation from its own throat into the throat of its playmate, and the result was fatal. Other means of transmission among children are the swapping of stick candies and the generous division of chewing-gum with their playfellows; in fact, the conditions are favorable with children for the transmission of the disease. With older persons there are not so many chances of contracting it, and in a great many cases it is difficult to trace it, the patient not having been anywhere where there was diphtheria, and it can only be accounted for by the theory that the bacilli must have been present on some food utensil, and getting into the throat, a proper soil for its growth was found, and the organism reduplicated to the extent of producing the disease.

This organism is a rod-shaped affair, one millimetre in size, and will grow on almost any media composed of animal material, but it does not grow readily upon dirt or other material outside of the body. It grows readily in bouillon, such as is served for soups, with peptones and salt added, and if we add sugar to that it grows more readily; but if we mix one part of bouillon and three parts of blood-serum we have delightful culture media, upon which this organism will grow with great celerity, particularly so if it is kept at the ordinary temperature of the human body, which is about 35° to 37° C.

In order to put the knowledge that we have to practical use, the health departments of various cities—New York being the first to adopt the method, Washington, Denver, Providence, and many other cities following—have taken up this method of diagnosing the presence of diphtheria by means of finding this particular organism in the throat. Of course, to do this with the patients all over the city, with the large number of physicians attending them, would seem to be impracticable; it would seem as if the technical knowledge required of the physicians for the manufacture and growth of this organism would not permit of its being carried into practical use, but the system has been so nicely developed that any physician who can succeed in seeing the back of a throat will be able to do as well as some other physicians in this matter of diagnosis. In order that physicians may have facilities for growing the bacteria of diphtheria, the boards of health in many cities have caused to be placed in drug-stores or other places in convenient localities this simple arrangement, which I will show you, where the physician may call and get it and use it according to the instructions whenever he wishes to make certain of his diagnosis in the case of a sore throat. It consists of an ordinary slate-pencil box, such as the children have, simple in construction, which is wrapped in waxed paper for two purposes, one being to prevent the evaporation of the moisture which is with the serum, and the second is to prevent, if possible, the contamination which is contained in the box being disseminated to the messenger or to any one else touching the box. The box contains one tube of this prepared serum, which is composed of two-thirds serum and one-third bouillon. After this has been prepared it is placed in a steam sterilizer. That kills all organisms that we do not desire to grow, because when we come to make an examination of our culture we want to be sure that everything there came from the throat. The other organisms which are liable to be there if this precaution is not taken are the ordinary bacteria of decomposition which will be found in the slaughter-houses and wherever animal tissue is decaying. In order to get the growth on this serum, the circular of instruction directs that we shall take the swab, consisting of a piece of ordinary iron wire, around which is placed some absorbent cotton, and that swab is used for the purpose of swabbing the back of the throat, giving it a circular twist over the whole of the fauces and wherever there is any of this slimy material. A portion of that is caught on the swab, and the swab is then run lightly over the surface of serum. The swab and serum are then replaced in the box, which is carefully wrapped up and sent to the office of the board of health, together with the name of the patient from whom it was taken and the physician who made the examination. Proper record is there made, and the box is again sent to the bacteriologist, who is to grow the culture and make the examination and decide whether the germ of diphtheria is there or not.

To go back to the serum which the physician has sent in: it is placed in the incubator, which is kept at a temperature of 37° C. day and night, constantly, and under these conditions of a suitable media at the right temperature the organisms thrive and grow, and the result is that on the following morning there will be upon the surface of the serum some sort of a growth, some organisms, if the procedures have been carried out as directed, and that growth under a small, low-power lens will manifest itself in the form of little round dots. These dots are what we call colonies,—that is, wherever an organism has been placed on the serum it has gone to work and reduplicated itself and introduced a new colony. As it is estimated that the growth from one organism will amount to about a million in twenty-four hours, you would readily suppose that the colonies would be very large, and if they should continue to grow at that rate we would soon be forced off the face of the earth; but Nature has provided that there shall be a limit to this reduplication. Of course, if we congregate in rooms that are too small, we soon die from the effects of it, and so it is with these organisms,—when they grow faster than Nature desires, they kill each other out and the growth is checked.

The question now before us, when we find these little colonies on the serum, is, What have we here? Is it a benign bacillus or a malignant one,—a disease organism or an ordinary one? Taking a loop of platinum wire, a small portion of the surface of the serum with these colonies on it is scooped out and rubbed over the surface of an ordinary microscopical cover-glass; it is allowed to dry, and is then stained with a solution of alkaline methyl blue, and the sample is examined microscopically. Then comes the question, which is so important for the patient, for the physician, and for the health department to solve, Are those bacilli of the species known as the Klebs-Löffler? If they are, then the sample is marked "K.-L.," and the result is reported at once by telephone to the physician who sends in the culture, in order that he may avail himself of that knowledge in treating his patient, and for the purpose of using antitoxin if he so desires.

If we do not find the Klebs-Löffler bacillus, it is regarded as a favorable sign for the patient, but not a decisive one. There are several reasons why the diphtheritic germ may not show in the culture, even though it was really present in the throat. The physician in the case may not have reached the back of the throat; he may have contaminated the swab with some other organisms, whose growth may have prevented the growth of the Klebs-Löffler bacilli; he may have failed to rub the swab properly on the serum; so that while we consider that the finding of the Klebs-Löffler bacillus is positive evidence that the patient has diphtheria, yet we do not consider that the absence of that organism is positive evidence that the patient has not diphtheria, and many times a second trial will give us a pure culture of the organism that we are looking for.

Having determined that we have a case of diphtheria, our next step is to at once, without going into the detail any further, notify the local board of health, which immediately, through its inspector, proceeds to placard the house,—that is, to put a card on the door stating that there is diphtheria in the house,—and also to distribute circulars of instruction to the family of the patient and any others who may be in the house. There was formerly great objections to this,—it was supposed to be a stigma on the family; it warned people not to go near them, for they were dangerous; they felt that they had lost their social standing and that they were looked down upon by their neighbors. This objection we have succeeded in partly overcoming by diplomatic means,—by telling them that in permitting this card to be placed there they are protecting not only the public, but are protecting themselves from trouble. For instance, if the card is on the house, any one coming in there does so on their own responsibility, and they have no redress if they contract the disease; if the card is not on the house, a person who may have had occasion to visit the house and afterwards contracted the disease at some bargain-counter or in the street-car or in a restaurant may bring suit against the parties who had refused to keep the card in sight when it was known that there was a case of diphtheria in the house. The result of this argument is that the majority of people now prefer the protection instead of objecting to what they used to consider a restriction. I regret to say that it is not only the lay people who give us trouble in this respect, but there are many physicians who have not yet seen fit to accept these facts regarding the infection and progress of the disease. They insist that there is no diphtheria there if they do not find the false membrane. The effect of their taking this position has given a great deal of trouble; in the city of Providence four lawsuits have been brought against the authorities as a result of having placed placards on houses where the physician in attendance had declared that there was no diphtheria, he depending entirely on finding the white membrane. The health department depends entirely upon the culture, and if that shows that there are organisms there in the throat, even though the membrane may have disappeared, the card remains, and as a result there is contention by law,—in other words, it is science against ignorance,—which will have to be fought out before intelligent courts. We have in the hospital now a case which is an excellent example of this absence of the clinical evidence of the disease. The membrane has long since disappeared, but we have taken ten or twelve cultures from that throat, extending over a period of eight or nine weeks, and we have found the germ in every one, while others in the same ward have come and gone within the same period. There is a case on record in the Boston Hospital of a lady from whom it was very difficult to expel these organisms; she continued to have them from early in May until late in October. You can readily see what a source of danger such a person would be to the public if she went about as if in perfect health. There is very little fear of infection if the patient is at the hospital or is properly quarantined at home, except to the nurse. It is the time when they get well,—that is, when they feel well and no longer suffer from the constitutional effects of the disease, and they go about among people,—that is the time when they are more dangerous to the general public than when they are critically ill themselves.

Now, the peculiar feature of this disease, as well as of other diseases caused by various kinds of bacteria, is the fact that it is not the bacillus itself which does all this harm, but in the process of its growth it produces a poison, a toxin, and this toxin, being absorbed by the system, produces constitutional effects to the extent of causing death; or, on the other hand, to the extent of the system finally becoming able to resist the action of the toxin. Immediately upon this toxin taking effect upon a patient, an antitoxin begins to form, which is quite antagonistic to the growth of the organism and to the formation of the toxin; the constitution being strong and capable of resisting the action of the toxin until a sufficient amount of this antitoxin has been formed, the throat of the patient, which before was a very desirable soil for habitation and reproduction of the bacilli, now becomes distasteful to them, and the reproduction ceases. On the other hand, if the system is not able to resist the effects of this toxin, the reproduction goes on, and results in the death of the patient. Now, making use of that knowledge that we have of the growth and effects of this bacterium, we set about to see if we cannot find some means of counteracting it, something which we can use for the treatment of this disease. That is done by a series of experiments progressing from laboratory to animals and from animals to man. We take the organisms which we know to be pure diphtheria and put them into a bouillon media, and allow them to remain in an incubator for forty-eight hours, during which time a rapid reproduction goes on, and taking a certain portion of this culture, we inject it into a horse. We could obtain the same results from other animals, but the horse is used because it is larger, stronger, gives more serum, and is less prone to die during the operation. Within twenty-four hours after the culture is injected he has a slight chill and a slight fever. When he recovers from this, we introduce double the quantity of toxin, and go on repeating this until we can introduce quite a large amount of the toxin without producing this fever, and this is a matter not of one or two days, but of six, seven, or eight months. The horse at that time has produced within the blood a quality which neutralizes the effect of that particular toxin whenever introduced. We then consider him as immunized, and his blood has within it the antitoxin quality which is so antagonistic to the development of this bacterium; in other words, they refuse to grow on suitable soil if there is this quality in the blood. Our next step, then, is to extract the blood from the horse which has been thus immunized, under antiseptic precautions, of course, by tapping the jugular vein and drawing off one or two gallons at a time. This is allowed to stand in the ice-chest until the clot is formed, and the serum is then combined with a suitable preservative camphor, and carbolic acid seems to serve the purpose very nicely. Now, taking this material, it is used on smaller animals for the purpose of ascertaining its strength. Various animals may be used, but the guinea-pig seems to be the most serviceable for bacteriological work. If we inject the toxic material into the guinea-pig, he dies in twenty-four hours. If, however, after the toxin has taken effect, we inject the antitoxin, we find that the guinea-pig recovers. The question now is, Where do we get the balance? How do we know how much antitoxin is going to be required to neutralize a certain amount of toxin? What is the unit from which we can proceed? Of course, it has taken a great deal of investigation to learn that one-tenth of a cubic centimetre of antitoxin would neutralize the effect of a cubic centimetre of the toxin,—that is, the antitoxin serum neutralizes ten times its strength in toxin. But the antitoxin seems to have the effect of stimulating the system in such a manner as to check the growth of the organisms. It gives assistance to the leucocytes, which are the large, round, white cells which are in the blood, and which seem to have a police protection over the body. These leucocytes have the power of resisting whatever poisons may have entered or generated, whether chemical or bacterial; they endeavor to overcome the effects of these poisons and to expel them from the system. So long as the leucocytes have the power to resist or dispose of the harmful organisms, so long are we able to resist disease; but if the whole system is depressed, then the leucocytes do not respond so quickly and vigorously, and these organisms of various kinds gain a firm foothold, and the toxin which is given off during the process of their growth is absorbed into the system. If we succeed in getting in the antitoxin soon enough, before the nervous system has been saturated with the toxin, we assist these leucocytes in their battle with the organisms, and the patient becomes an uncongenial place for their growth.

We may be asked, How do you know that this antitoxin is good for the patient? We reply, Statistics have shown this to be the fact. But statistics may be used in various ways. The figures of reliable observers show that the mortality, which was perhaps thirty to thirty-five per cent. of the total number of cases before the use of antitoxin, has decreased, until it is now claimed that but about seven per cent. of deaths occur where antitoxin is used. The argument is also raised by the other side that it is not a virulent epidemic; that under certain climatic conditions the organism has a more severe action. Personally, I do not rely so much upon statistics as I do upon actual observation, for when you are called to your patient, you find the trachea obstinately producing a whistling sound, gasping for breath, the throat tumefied to the extent that it almost prohibits the passage of air to the lungs, and covered with excretions, with that dreaded membrane, and in one night after the injection of the antitoxin the changes occur, and on your next visit the patient may be respiring freely, the slimy material has disappeared, and the tumefaction has subsided, leaving usually that white, tenacious, leathery, tough membrane standing out in its true character. This we consider the most effective argument for our present system of diagnosis and treatment of diphtheria, and where it is of especial value to us is in determining the character of those cases where there are no symptoms present, the liability being so great in such cases of transmitting the disease to others. The importance of this was never understood until recently. After a case of diphtheria had recovered, we went through the usual process of fumigating, and thought we had completely eradicated the disease from that house; but in a couple of weeks we had another case in the same house, and we could not understand how it happened. We have now followed the matter up, so that the law of the city of Providence requires that when a child has had diphtheria and desires to return to school, it shall be shown that there are no diphtheria bacilli in the child's throat, nor in the throat of any member of the family. It has entailed lots of work, but it has taught us a great deal. We have a pretty good control of the disease when patients are admitted to the hospital. They are not allowed to go from there until we have obtained negative cultures on three consecutive examinations. That places us in a very certain position with regard to the patients' condition when they leave the hospital, and our experience in this matter has shown the value of this precaution, for of the number of cases examined as precautionary about twelve per cent. showed the presence of the diphtheria bacillus for several days after the patients appeared to be fully recovered. Now, if that twelve per cent. had been allowed to go free, it is impossible to estimate the number of cases which might have resulted, though the original patients would probably not have succumbed to the disease.

In the use of the antitoxin the method which is employed is to use a quantity which we think is equal in proportion to the weight of the person requiring it, and that amount runs all the way from one thousand to two thousand antitoxin units. The usual method of introduction is by means of an ordinary syringe, such as is used for drawing off pus from a cavity, and the cylinder used to be capable of containing about five cubic centimetres; but now they have succeeded in immunizing horses to such an extent that two cubic centimetres of serum will do what ten used to do. The place selected is usually a place where a fold of the skin can be caught up between the thumb and forefinger,—by some, between the scapulæ on the back; by some, on the thigh. The place which I select is the side of the abdomen just below the ribs. The skin here is less tense and can be easily grasped, and if there is a movement of the patient, less motion is found than nearer the extremities. Though the pain to the patient is nothing more than would result from a pin-prick, there is usually a good deal of scratching and struggling and sometimes opposition on the part of the family, by whom it is thought that the injecting of something under the skin must be more dangerous than putting things down the throat.

Now, one other point: In regard to the question of what is to be done, after the patient gets well, with the clothing, furniture, eating utensils, handkerchiefs, and anything in the room which may have received the bacillus from the patient. Of course, it is quite a puzzle to know what to do so that we may feel certain that the room and articles used by the patient may be used by other people without danger of contracting the disease. All the clothing, bedding, and anything removable are usually put in a steam sterilizer, and for the nicer articles we now have hot-air cylinders, where these articles are allowed to remain for one to two hours at a very high temperature,—say 250° C.,—and this intense heat will kill all germ-life, but does not injure the articles. A fine laundered shirt may be put in there, and it will come out in as good condition as it went in. But the conditions in the room, of course, still remain, and how to get into every nook and crevice of that room and make sure that we have destroyed all the bacteria has always been a source of anxiety to us. We used to go into a room with a pound of sulphur for every thousand cubic feet of space and set fire to it. and think we had done considerable, but we learned through our investigations in bacteriology that we had really accomplished very little, for, upon taking a culture of the diphtheria bacilli which had been exposed to the fumes of the sulphur the usual length of time, we found that it could grow, showing that the sulphur had no effect whatever. In addition to that, the sulphurous acid would tarnish everything unlacquered in the room and destroy the clothing, if moist. Chlorine is of no value, because it rots everything, so that for a time we were obliged to do our house-cleaning with bichloride, a solution of, say, one five-hundredth, and do the best we could; but we have recently discovered, again through the work of bacteriologists, a germicide which will destroy not only diphtheria organisms, but every other organism of disease at present known. It is by means of a simple flame from wood alcohol which passes through a sieve made of asbestos and platinum. The vapor of the wood alcohol, passing through a heated sieve, produces formaldehyde, and that, escaping into the room in certain quantities, will saturate every article there and penetrate into every crack or corner; but it must be introduced into the room in a rapid manner and in sufficient quantity, when it succeeds in killing the organisms after an exposure of four or five hours, and upon that we shall rely, probably, in the future for sterilizing, until some more effective germicide is discovered.

You must pardon me for taking up so much of your time. I hope I have not destroyed your confidence in selecting a speaker not exactly in your own profession and a subject not exactly in your line, but, as I said, it is a subject which is fraught with great interest not only to the health department but to the entire community, and I am very glad to find that you have taken an interest in it.

